# Face-to-Face Psychiatry Placements and Their Impact on Student Attitudes to Psychiatry

**DOI:** 10.1192/bjo.2022.156

**Published:** 2022-06-20

**Authors:** Abigail Swerdlow, Sonya Rudra

**Affiliations:** 1 East London NHS Foundation Trust, London, United Kingdom; 2 Queen Mary University of London, London, United Kingdom; 3 Tavistock and Portman NHS Foundation Trust, London, United Kingdom

## Abstract

**Aims:**

Last year the COVID-19 pandemic meant that there could not be any face-to-face psychiatry placements for medical students at QMUL (Queen Mary University of London). This year there has been a return to face-to-face placements within psychiatric settings. The aim of this project was to evaluate whether face-to-face placements have an impact on medical student attitudes to psychiatry. This will have implications for recruiting students into the specialty, once they qualify.

**Methods:**

128 students were placed in face-to-face psychiatric settings at the beginning of their 4th year of medical school. The placements were 3 days a week for 5 weeks. The placements varied with some students being placed in inpatient services and others within the community, across a broad range of subspecialties including child and adolescent, general adult and forensic. Students were given the Attitudes To Psychiatry Questionnaire to fill out before and after their placement. Students were also given the opportunity to provide open text feedback on their placement in the form of a weekly feedback form. Results were analysed using simple descriptives of data and paired t-tests. The study was conducted with permission from Associate Dean for Undergraduate Teaching and QMUL Centre Lead for Psychiatry.

**Results:**

115 students (89.8%) completed pre-placement attitudes to psychiatry questionnaire and 51 students (39.9%) completed the post-placement questionnaire.

Paired t-tests were used to compare average pre and post-placement results for individual questions. There were significant changes in student responses to questions about psychiatric undergraduate training being valuable, attitudes to psychiatrists and psychiatric treatment. Lots of students gave positive feedback on their placements citing interesting experiences, helpful seniors and varied learning opportunities.

**Conclusion:**

Students having face-to-face psychiatry placements has objectively changed some attitudes to psychiatry and is very important for their experience and interest in the specialty. The students have valued the exposure and contact with patients and the varied experiences. This will hopefully lead to more students considering psychiatry as a career as well as keeping mental health as a priority for any patient that they see.

